# Update on the diagnosis and management of neurocysticercosis

**DOI:** 10.1590/0004-282X-ANP-2022-S115

**Published:** 2022-08-12

**Authors:** Osvaldo Massaiti Takayanagui, Tissiana Marques de Haes

**Affiliations:** 1 Universidade de São Paulo, Faculdade de Medicina de Ribeirão Preto, Departamento de Neurociências e Comportamento, Ribeirão Preto SP, Brazil.

**Keywords:** Cysticercosis, Taenia solium, Epilepsy, Magnetic Resonance Imaging, Cerebrospinal Fluid, Albendazole, Praziquantel, Cisticercose, Taenia solium, Epilepsia, Imageamento por Ressonância Magnética, Líquido Cefalorraquiano, Albendazol, Praziquantel

## Abstract

**Background:**

Neurocysticercosis (NCC) is a serious public health problem in several developing countries, including those in Latin America, Asia, and Africa. NCC is considered to be the main cause of late-onset epilepsy in endemic areas.

**Objective:**

This review summarizes recent advances in diagnosis and therapy of NCC. **Methods:** Relevant articles and books were reviewed and used as a source of information for this review.

**Results:**

The diagnosis of NCC is based upon neuroimaging studies (MRI and computed tomography) and laboratory analysis of the cerebrospinal fluid (CSF). Praziquantel and albendazole are considered parasiticidal drugs against NCC, but there is an intense debate over the value and safety of these drugs.

**Conclusion:**

Given the relative scarcity of clinical trials, more comparative interventional studies, especially randomized controlled trials in long-term clinical evolution, are required in order to clarify the controversy over the validity of parasitic therapy in patients with NCC.

## RESUMO

**Antecedentes:**

A neurocisticercose (NCC) é grave problema de saúde pública nos países em desenvolvimento, especialmente na América Latina, Ásia e África. A NCC é considerada a principal causa de epilepsia de início tardio nas regiões endêmicas. **Objetivo:** Este artigo pretende discutir os recentes avanços no diagnóstico e tratamento da NCC.

**Métodos:**

Artigos científicos e livros relevantes serviram de fonte de informação para esta revisão.

**Resultados:**

O diagnóstico da NCC é fundamentado nos exames de neuroimagem (ressonância magnética e tomografia computadorizada) e do líquido cefalorraquiano (LCR). Atualmente, praziquantel e albendazole são considerados eficazes na terapêutica etiológica da NCC, mas há intenso debate quanto à validade e segurança desses medicamentos.

**Conclusão:**

Pela relativa carência de ensaios clínicos, são necessários novos estudos particularmente randomizados, controlados e com análise de desfechos clínicos a longo prazo para o esclarecimento da polêmica envolvendo a validade da terapêutica parasiticida na NCC.

## INTRODUCTION

Neurocysticercosis (NCC), the central nervous system (CNS) infection caused by the larval form of *Taenia solium*, is a serious public health problem in several developing countries, including those in Latin America, Asia, and Africa. NCC is considered to be the main cause of late-onset epilepsy in endemic areas^1^ ^,^ ^2^. Dangerous sanitary and poor socioeconomic conditions combine to perpetuate its dissemination. 

The prevalence and mortality of NCC are probably grossly underestimated because diagnosis requires neuroimaging (computed tomography and/or MRI) which is largely unavailable in highly endemic regions^1^.

Human cysticercosis is the result of accidental infection with the embryonic form due to the ingestion of water or food contaminated with *Taenia solium* eggs. 

## PATHOLOGY

**Figure 1.**
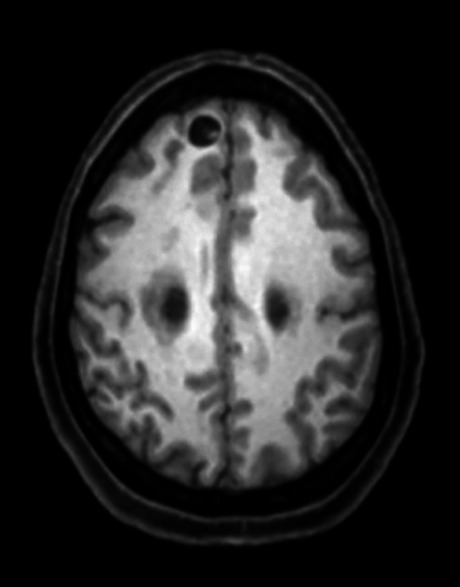
MRI intraparenchymal viable cysticercus with scolex.

**Figure 2.**
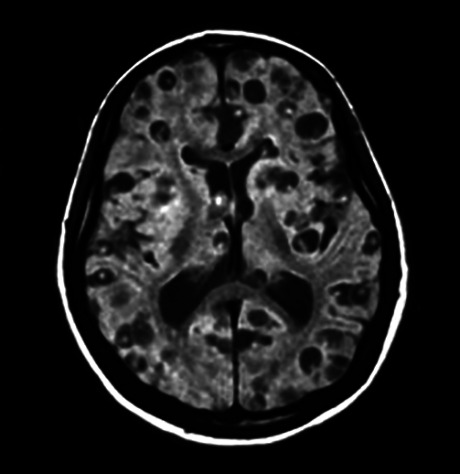
MRI multiple intraparenchymal viable cysticerci.

**Figure 3.**
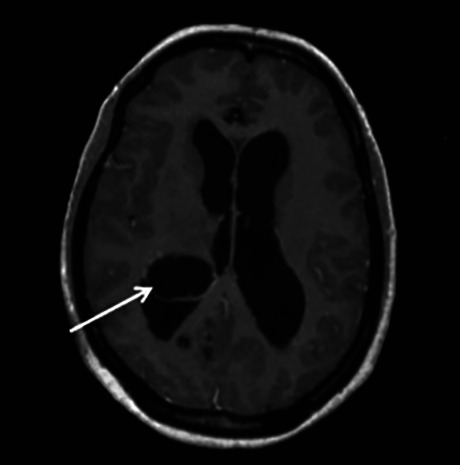
MRI intraventricular cysticercosis.

The cysts located in the brain parenchyma may range in size from a few millimeters to several centimeters. Initially, viable cysticerci have a translucent whitish membrane, clear vesicular fluid, and an invaginated larva, the scolex, with minimal surrounding inflammation (vesicular stage)^3^ ^,^ ^4^ (Figures 1 and 2). The cysticerci developing in the ventricles (Figure 3) or in the subarachnoid space usually reach a larger size and often take on the form of racemose cysticerci, which are characterized by a membrane of irregular thickness and the absence of a scolex. These are usually clustered into multiple interconnected vesicles, resembling a cluster of grapes^3^ ^,^ ^4^. 

The cysticerci located in the brain parenchyma undergo a natural evolutionary process which culminates with their degeneration within a period of approximately three to six years. Replacement of the clear fluid with a jelly-like whitish material occurs and the parasite is surrounded by host inflammatory cells (colloidal stage). At a more advanced stage, the cyst begins to be reduced in size, the walls become thicker and its contents, due to mineralization with calcium salts, are transformed into coarse granules (granular stage). The final stage is when the cyst attains complete mineralization (calcified nodular stage)^3^.

## CLINICAL FEATURES

The clinical manifestations of NCC largely depend on the number, type, size, localization, and stage of development of cysticerci, as well as on the host immune response against the parasite. There are no pathognomonic features of a typical NCC syndrome^5^ ^,^ ^6^.

Intraparenchymal NCC is usually associated with a good prognosis. Frequently, patients with few intraparenchymal cysts remain asymptomatic^7^, although some patients develop seizures. On the other hand, in patients with massive cerebral infection, uncontrolled seizures and cognitive deficit may develop^5^.

Seizures are widely reported to be the most common symptom, occurring in 70-90% of patients and NCC is considered to be the main cause of late-onset epilepsy in endemic areas^1^. Partial seizures, with or without secondary generalization, predominate in most cases. Seizures are thought to occur from parenchymal irritation because of active inflammation occurring with the death of the cysticercus or from gliosis associated with end-stage calcified lesions. In many patients, as the cysticercus calcifies, seizures tend to become less frequent although patients usually require continuous treatment with anti-seizure medication. 

When cysticerci lodge within the ventricular system a life-threatening acute intracranial hypertension secondary to hydrocephalus may develop, directly related to obstruction of the flow of CSF by the cyst or by inflammatory

reaction of the ependyma. Although the cysts may be found anywhere within the ventricular system, the fourth ventricle is most commonly involved^8^. 

Acute intermittent hydrocephalus, with violent headaches, attacks of positional vertigo or loss of consciousness induced by abrupt movements of the head (Bruns’ syndrome), or even sudden death, may result if a mobile ventricular cyst is present^4^.

**Figure 4.**
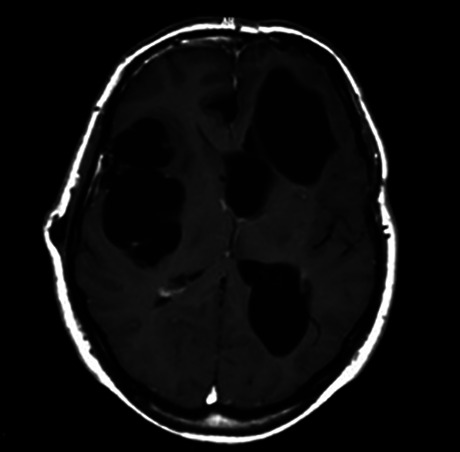
MRI multiple giant cysticerci.

**Figure 5.**
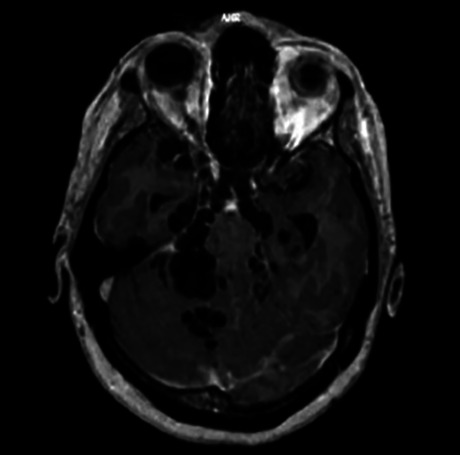
MRI subarachnoid racemose cysticercosis, involving basal cisterns.

Cysts in the subarachnoid space may invade the Sylvian fissure and grow to large sizes, reaching several centimeters in diameter (giant cysts) (Figure 4), causing intracranial hypertension with hemiparesis, partial seizures or other focal neurological signs. Subarachnoid cysts may also invade the basal cisterns; initially the growing membranes resemble a bunch of grapes, hence this form of disease is called “racemose” cysticercosis (Figure 5). It is associated with an intense inflammatory reaction, fibrosis and progressive thickening of the leptomeninges at the base of the brain. In approximately 50-60% of the cases, there is an obstruction of the CSF circulation, resulting in hydrocephalus and progressive intracranial hypertension and mortality over 20% of cases^4^. Signs of arachnoiditis, cognitive and psychiatric dysfunction, cranial nerve palsy, chiasmatic syndrome, cerebellopontine-angle syndrome, and cerebral infarcts secondary to occlusive endarteritis may develop^8^ ^-^ ^11^. When hydrocephalus secondary to cysticercotic meningitis is present, mortality is high (50%), and most patients die within two years after CSF shunting^12^. Therefore, ventricular and basal cisternal locations are considered to be malignant forms of neurocysticercosis^13^ .

Intracranial hypertension also occurs in patients with cysticercal encephalitis as the result of a massive infection of the brain parenchyma inducing an intense immune response and diffuse brain edema^14^. 

Some patients with NCC present with psychiatric and cognitive impairment^15^ ^,^ ^16^.

The natural history of NCC is largely unknown and most data are based on retrospective and uncontrolled studies, mainly from neurological hospital settings. Prospective and properly designed studies are keenly awaited to help clarify the natural history of the disease^5^. 

## DIAGNOSIS

The diagnosis of NCC is based upon neuroimaging studies and antibody/antigen detection in the serum and the CSF.

Neuroimaging with either computed tomography (CT) scan or magnetic resonance imaging (MRI) is considered the gold standard for diagnosis of NCC. Early in the infection, a viable cyst appears as a spherical hypodense lesion on CT scan and as a CSF-like signal on MRI. Both CT and MRI are able to show the invaginated scolex. In the degenerative phase, the cyst shows a ring-like or a nodular contrast enhancement, with or without perilesional edema. A final stage is observed when the cyst dies and a process of mineralization and resorption takes place, resulting in a calcified nodule.

Since the cyst membrane is thin and the fluid is isodense within the CSF, non-inflamed extraparenchymal (ventricular or subarachnoid) cysticerci are usually not visible on CT and may only reveal subtle, indirect findings on MRI scans.

MRI is more sensitive than CT scans for the diagnosis of NCC since it improves recognition of the perilesional edema and degenerative changes of the parasite, as well as small cysts or those located inside the ventricles, brain stem, cerebellum and the racemose vesicles at the level of the posterior fossae and basal cisterns. However, CT scans are more sensitive in the detection of calcifications.

Recently, the development of three-dimensional MRI sequences, such as Fast Imaging Employing Steady-State (FIESTA) and 3D constructive interferences steady state (3D CISS) has improved the sensitivity and specificity of MRI, especially for subarachnoid and ventricular cysticerci^17^ ^-^ ^19^

Analysis of CSF samples is an important parameter for the assessment and follow-up of patients with a suspicion of NCC. The most frequent CSF alterations are mononuclear pleocytosis and the presence of eosinophils and specific antibodies detected by enzyme-linked immunosorbent assay (ELISA) or enzyme-linked immunoelectrotransfer blot assay (EITB). The presence of pleocytosis and specific antibodies coincides with the degenerating stage of cysticerci and intensification of the host immune-inflammatory response^4^. 

A number of tests have been developed for the detection of antigens and anti cysticercal antibodies in CSF. Although enzyme-linked immunosorbent assay (ELISA) and enzyme-linked immunoelectrotransfer blot assay (EITB) in CSF have a high level of sensitivity and specificity, major concerns for the use of EITB are its complexity, time of execution, and cost^20^ ^,^ ^21^. *Taenia* antigens may also be detected in the CSF specially in the clinically active forms of NCC, being a more sensitive marker than the classic eosinophil presence^22^. 

Immunodiagnostic tests of serum samples have been widely used in diagnostic and epidemiological studies of cysticercosis. The EITB assay is almost 100% sensitive for patients with either multiple active parenchymal cysts or extraparenchymal NCC. However, the sensitivity is lower for patients with either single parenchymal cysts or calcifications alone. New assays are being developed. Studies of monoclonal antibody-based ELISA tests for detection of antigens have shown detection of circulating and CSF antigen and may be useful for both diagnosis and post therapeutic monitoring, with a correlation between circulating antigen detection and CT scanning results during follow-up^23^ ^,^ ^24^. Other assays for detection of parasite antigen are under development including polymerase chain reaction^25^, quantitative PCR^26^, recombinant antigens^27^ and a combination of EITB banding patterns with antigen-ELISA^28^.

Because clinical manifestations are pleomorphic, most neuroimaging findings are not pathognomonic, and several immunological tests show low levels of sensitivity and specificity, some authors have proposed diagnostic criteria for diagnosis NCC^29^ ^-^ ^31^. 

Unfortunately, diagnosis of NCC requires neuroimaging techniques (CT and/or MRI), immunodiagnostic tests in serum (EITB) and CSF (antibodies and/or antigens) that are not readily available in many setting where the disease is prevalent^1^, due to resource constraints^19^.

## TREATMENT OF NCC

The treatment modalities available to patients with NCC include surgery, symptomatic therapy and antiparasitic drugs.

### Surgery

Prior to the advent of anti cysticercal drugs, surgery was the primary therapy for NCC - mainly open surgery for excision of large cysts or cysts in the ventricles. The role of surgical therapy in the management of NCC has significantly decreased over time and surgery is now mainly restricted to placement of ventricular shunts for hydrocephalus secondary to NCC and for occasional cases of accessible intraventricular or racemose subarachnoid cysts, mainly by endoscopic approach^32^. 

### Symptomatic therapy

Symptomatic therapy is probably more important in NCC than in any other infectious disease^33^. 

Most patients with NCC present seizures and the administration of standard doses of a single-first-line anti-seizure medication such as phenytoin or carbamazepine usually results in adequate seizure control. The optimum length of anti-seizure medication therapy has not yet been determined, but it has been suggested that it should be continued until serial neuroimaging studies show resolution of acute lesions^34^. After disappearance of the cysts, most patients who have been free of seizures for two years can eventually discontinue anti-seizure medication.

Since inflammation is the conspicuous accompaniment in most forms of NCC, corticosteroids represent the primary form for attenuating the inflammatory reaction that may cause severe recurrent seizures, focal neurological symptoms and intracranial hypertension syndrome. Additionally, corticosteroids are fundamental for patients with cysticercal encephalitis, arachnoiditis and angiitis. Only scarce controlled data exist to determine when and what type of corticosteroids and the treatment regime to use. The most frequent regimen is dexamethasone at doses of 4.5 to 12 mg/day. Prednisone at 1 mg/kg/day, daily or three times a week, may replace dexamethasone when long-term steroid therapy is required. For patients who develop chronic or recurrent cerebral inflammation, methotrexate may be useful as a corticosteroid-sparing or replacement agent^35^. 

Symptomatic treatment also includes the placement of ventricular shunts for hydrocephalus associated with intracranial hypertension syndrome.

### Anti Cysticercal Drugs

Therapy for NCC, formerly restricted to palliative measures, has advanced with the advent of two drugs considered to be effective: praziquantel (PZQ) and albendazole (ALB). However, pharmacologic therapy should not be used indiscriminately in all cases but individualized on the basis of clinical syndrome, characteristic of the cysts, and the host immune response. The precise indication is for symptomatic patients showing multiple viable brain parenchymal cysticerci. The viability of cysticerci is characterized by the presence of rounded areas of hypodense lesions on CT scans with a scolex inside the cyst, better shown on MRI, without contrast enhancement or surrounding edema^1^ ^,^ ^36^ ^-^ ^39^.

The goal of anti cysticercal therapy is the simultaneous destruction of multiple cysts and then controlling the resulting inflammatory reaction with steroids. This strategy of preventing prolongation of brain inflammation due to degeneration of multiple cysts at different times would allow better clinical evolution than the natural progression of NCC^37^ ^,^ ^38^.

*5.3.1 Praziquantel (PZQ)*

Praziquantel is an acylated isoquinoline-pyrazine with broad anthelmintic activity and strong activity against schistosomes and cestodes.

Although its exact mechanism of action is not fully understood, it is generally accepted that PZQ changes metabolism and intracellular calcium with the main effect of inhibition of muscular movements^40^. The main PZQ-metabolite in plasma is Trans-4-hydroxy praziquantel formed by cytochrome P450^41^.

The effective dosage used in most studies was three divided doses of 50 mg/kg/day for two weeks.

*5.3.2 Albendazole (ALB)*

The benzimidazole derivate ALB is a broad spectrum anthelmintic drug that affects the dynamics of vesicular traffic^42^.

In human liver microsomes, ALB is oxidized to the active albendazole sulfoxide (ASOX) metabolite by flavin monooxygenases and the cytochrome P450 system. The concentration of ASOX varies widely among individuals and the drug has a half-life of six to 15 hours. ASOX crosses the blood-brain barrier and its concentration in CSF varies as a pharmacokinetic characteristic of the drug. 

The ALB dosage regimen currently used is 15 mg/kg/day divided into two doses every 12 hours for 1 week. 

*5.3.3 Side effects*

Between the second and fifth days of the antiparasitic therapy, there is usually an exacerbation of neurological symptoms, such as headache, vomiting and seizures in 50-80% of the patients, accompanied by exacerbation of pleocytosis in the CSF in 50-75% of cases^43^ ^,^ ^44^. These reactions are eliminated or minimized by increasing the dose of dexamethasone. This is probably not caused by a toxic reaction to the drugs, but rather is an inflammatory reaction produced by the host in response to the death of the parasite^36^ ^,^ ^37^ ^,^ ^38^ ^,^ ^44^.

Occasionally, severe reactions such as brain infarction and death due to acute intracranial hypertension syndrome may occur^44^. Although severe decompensation of intracranial hypertension is a rare occurrence during treatment, this possibility advises against treatment on an outpatient basis, especially for patients with massive infection, ventricle cysts and subarachnoid racemose cysticercosis. Additionally, these severe complications support the recommendation of the simultaneous use of dexamethasone in these cases. However, it should be kept in mind that coadministration of dexamethasone reduces plasma levels of PZQ. Therefore, some authors have recommended reserving steroids as symptomatic treatment for patients who experience headache or vomiting during PZQ therapy for intraparenchymal lesions.

On the other hand, dexamethasone should not cause concern with ALB therapy since simultaneous administration increases plasma levels of ASOX by slowing the rate of its elimination^45^ ^,^ ^46^. 

*5.3.4 Albendazole versus Praziquantel*

Most comparative investigations have shown that ALB is more effective than PZQ in reducing the number of cysts and in inducing overall clinical improvement, with a lower frequency of adverse reactions. However, most of these trials have been uncontrolled, observational imaging studies and none of them was designed to evaluate seizure control. The meta-analyses of comparative trials suggested that ALB is more effective than PZQ regarding clinically important outcomes in patients with NCC^47^ ^,^ ^48^. 

ALB is considered to be the drug of choice for therapy of NCC because of its widespread availability and low cost. ABZ has also been shown to be more effective than PZQ, probably because ABZ penetrates into the CNS more efficiently^45^ ^,^ ^49^. Nonetheless, the effectiveness of these drugs as single antiparasitic agents is only partial, with approximately 60% of parasites destroyed and only 35% of patients being free of surviving cysts after a first round of treatment^50^ ^,^ ^51^.

*5.3.5 Albendazole plus Praziquantel*

Recent studies have demonstrated an improved cysticidal effect of the simultaneous administration of albendazole and praziquantel, primarily in patients with more than two cysticerci^52^ ^,^ ^53^. This schedule is based on the observation that serum levels of Albendazole sulfoxide (the active metabolite of ALB) are variable, but levels are higher and more consistent when the two drugs are combined^54^ ^,^ ^55^.

The combined schedule is 15mg/kg per day for ALB and 50mg/kg per day for PZQ, divided in two or three daily doses while the proposed length of treatment ranges from one to two weeks for parenchymal and ≥ 1 month for subarachnoid lesions^1^ ^,^ ^19^

*5.3.6 Controversies over anti cysticercal therapy*

Anti cysticercal therapy has been marked by intense controversy. The descriptions of spontaneous resolution of parenchymal cysticercosis with benign evolution, risks of complications and reports of no long-term benefits have reinforced the debate over the usefulness and safety of anti cysticercal therapy.

Most available data describing the effectiveness of anti cysticercal treatment are from uncontrolled studies with a significant selection bias. Many studies have documented that antiparasitic therapy results in death and resolution of viable cysts, but the clinical benefit of this treatment has been questioned. Randomized controlled trials evaluating the clinical benefit of treatment have yielded conflicting data with some studies indicating a benefit and others failing to show any difference^56^. 

A randomized, double-blind, placebo-controlled trial showed that patients who received ALB had fewer generalized seizures, although there was no significant difference in the overall number of seizures or the number of patients with seizures^57^. 

A systematic review by the Cochrane Collaboration concluded that for patients with a single cyst, there was less seizure recurrence in the albendazole group compared to the placebo/no anthelmintic group, but it was uncertain whether albendazole reduces seizure recurrence of those with multiple cysts while albendazole probably increases radiological clearance and evolution of lesions^58^. 

*5.3.7 Failure of anti cysticercal therapy: possible explanations*

The discrepant data over the efficacy of anti cysticercal therapy may be explained by several factors. 

First, there is considerable inter-individual variability in the plasma concentration of PZQ and ASOX^45^ ^,^ ^59^ ^,^ ^60^. The exact mechanism of this variability in the plasma concentration of the drugs is not clear but may include gender factor^61^ - greater ASOX concentration in women than in men - and the low solubility of ALB in the gastrointestinal tract fluid and the high presystemic elimination^62^. 

Second, there are important food and drug interactions. Dexamethasone decreases plasma concentration of PZQ but increases ASOX^45^ ^,^ ^46^. Food, a high carbohydrate diet, grapefruit juice and cimetidine increase plasma PZQ concentration^40^ ^,^ ^63^ ^-^ ^65^. On the other hand, antiepileptic drugs significantly reduce the plasma concentrations of both PZQ and ASOX^66^. Therefore, it was suggested that higher doses of ALB would be necessary when the agent is co-administered with antiepileptic drugs.

Third, ASOX is a chiral metabolite. After intestinal absorption, ALB is rapidly converted into the active metabolite ASOX, a mixture of (+)-ASOX and (-)-ASOX enantiomers^67^ ^-^ ^69^. Plasma concentrations of the (+)-ASOX enantiomer are approximately nine times higher than those observed for the (-)-ASOX antipode^59^. ASOX is also found in the CSF at a high proportion (a 2:1 serum to CSF ratio)^45^. It demonstrated an accumulation of the (+)-ASOX metabolite in the CSF, which was three times higher than that of the (-) antipode^70^ ^,^ ^71^. The clinical significance of these findings remains to be tested. Although ALB is useful for therapy of cysticercosis in the subarachnoid space where CSF (+)-ASOX and (-)-ASOX concentrations would be of paramount importance, ALB is more effective in brain parenchymal cysticercosis^36^. However, due to the difficulty in measuring ASOX enantiomers within the brain parenchyma, CSF sampling is the best substitute for CNS drug penetration. In addition, there are no data in published studies over which ALB metabolite (+)-ASOX, (-)-ASOX or both, is actually effective or the respective concentrations required for proper treatment of NCC. Future studies concerning CSF enantiomer concentration and the clinical outcome of NCC would clarify the question over the therapeutic role of each ALB metabolite against the cysticerci.

Similarly, PZQ is also a racemic mixture of two stereoisomers, of which only the (-)-(R)-isomer possesses activity against schistosomes. Trans-4-hydroxy praziquantel, the major PZQ metabolite, is also a chiral compound and the activity seems to be related to the (-)-(R)-isomer. PZQ metabolism is enantioselective with accumulation of the (+)-(S)-PZQ and (-)-(R)-4-OHPZQ in human plasma^72^. To date, there is no study that analyzes the plasma concentration of PZQ enantiomers during treatment of human NCC.

Given that multiple drug therapy is a common therapeutic practice in patients with NCC, careful evaluation of drug interactions is essential in understanding the actual effectiveness of anti cysticercal drugs^73^. On the basis of the high inter-individual variability and the complex pharmacological interactions, monitoring of plasma concentration of PZQ and ASOX would be highly recommended in patients receiving anti cysticercal therapy.

### Management of NCC according to its location

*5.4.1 Parenchymal brain cysticercosis*

The weight of a vast amount of literature showing disappearance of brain parenchymal cysticercosis with subsequent clinical benefits should not be overlooked. A few studies supporting the argument that ALB does not modify the clinical outcome have shown persistence of lesions on neuroimaging studies as a factor for recurrence of seizures. The paucity of adequate therapeutic trials is in fact an irrefutable argument but considering the efficacy, good tolerance and the risks of leaving multiple cysts dying at different times, anti cysticercal therapy should be considered in every patient with multiple viable intraparenchymal cysticerci. 

Nowadays, ALB is the medication of choice for the treatment of symptomatic patients presenting multiple viable cysts in the brain parenchyma on neuroimaging studies. Simultaneous administration of ALB plus PZQ is an alternative schedule^52^ ^,^ ^53^. The Infectious Diseases Society of America (IDSA) and the American Society of Tropical Medicine and Hygiene (ASTMH) recommend ALB monotherapy for patients with one to two viable parenchymal cysticerci and ALB combined with PZQ for those with more than two viable parenchymal cysticerci^19^. Concomitant steroids are recommended, particularly dexamethasone^38^ ^,^ ^46^. However, the optimal drug, dose, and duration have yet to be defined^74^. 

ALB is not usually recommended for single enhancing lesions (SEL), given that these lesions correspond to degenerating cysticerci and are usually resolved spontaneously within a couple of months, regardless of therapy^36^ ^,^ ^38^ and requires only symptomatic treatment (anti-seizure medication - ASM, steroids, etc.). Additional corticosteroid treatment was found to have a beneficial effect both on seizure reduction and cyst resolution ^[^ ^75^ ^]^.

Calcified lesions do not require antiparasitic therapy because the cysticerci are thought to be already dead, even when remnants of scolex are detected by chance. Perilesional edema and contrast enhancement around calcifications in patients with seizures activity may require transitory anti-inflammatory medication^36^.

*5.4.2 Extraparenchymal cysticercosis*

Extraparenchymal cysticercosis (subarachnoid, intraventricular and racemose form) is associated with a poor prognosis and requires a more aggressive approach. When feasible, complete surgical excision of lesions remains the definitive therapy. In patients with hydrocephalus or intracranial hypertension, the priority is to manage the hypertension problem before considering any other form of therapy. Hydrocephalus, whether caused by ventricular or cisternal cysts, usually require ventricular shunting which may be complemented with excision of approachable ventricular or cisternal cysts.

Ventricular freely mobile cysts should be treated with surgical or, preferably, neuroendoscopic removal^9^ ^,^ ^10^ ^,^ ^36^. There are, however, contraindications to endoscopic cyst removal. Inflamed or degenerating cysticerci are frequently adherent to the ventricular walls and ependymal and attempted removal of adherent cysts is associated with a high risk of hemorrhage and neurologic sequelae^19^. As antiparasitic drugs may induce cyst inflammation, pre-operative antiparasitic therapy should generally be avoided.

The optimal treatment of subarachnoid giant cysts with local compression or mass effect is surgical-cyst excision via a direct approach, or at least cyst evacuation and partial resection via a direct or stereotaxic approach. However, in patients with racemose cysticercosis, complete excision of all cysts in the basal cisternal is usually impracticable. Most experts have concluded that there are benefits of cysticidal therapy, but that more intensive therapy may be needed that is traditionally used for parenchymal disease. A long course (at least a month) of ALB is indicated in association with dexamethasone^75^. Other approaches have included high-dose ALB (30 mg/kg/day), repeated courses of ALB, PZQ (50 mg/kg/day for 30 days) and sequential or combined use of both drugs^76^. Inflammation plays a key role in the pathogenesis of subarachnoid neurocysticercosis and nearly all of the complications (chronic meningitis, vasculitis and hydrocephalus) are the result of an inflammatory reaction to parasite antigen. Thus, concomitant corticosteroid therapy is essential to avoid complications due to the ensuing inflammation in the subarachnoid space especially after the administration of antiparasitic drugs, with a careful tapering schedule to avoid cerebrovascular complication and hydrocephalus^19^.

## FUTURE PERSPECTIVES

Adequate experimental models of NCC need to be developed to test the efficacy of the available and new drugs and vaccines for NCC. 

Due to high inter-individual variability and complex pharmacological interactions, monitoring of plasma concentration of ASOX and PZQ will be highly recommended in future trials. The determination of minimal plasma concentrations of ASOX and PZQ, which is effective against cysticerci, would allow the optimization of therapy by monitoring the plasma concentration and dose adjustment during treatment. 

Investigations into which ALB metabolite, (+)-ASOX, (-)-ASOX or both, is actually effective against cysticerci and the respective concentrations required for correct treatment are also needed. In the future, studies concerning CSF enantiomer concentration and clinical outcome of NCC will be able to clarify the question over the therapeutic role of each of the ALB metabolites against the cysticerci.

Additionally, asymmetric synthesis or purification of ASOX enantiomers, (+)-ASOX and (-)-ASOX, might allow the separate evaluation of the efficacy of each one of them in experimental models or in vitro studies. 

Similarly, the role of PZQ stereoisomers should be evaluated in future clinical trials. In addition, the pharmacological activity of trans-4-hydroxy praziquantel, the major PZQ metabolite which is also a chiral compound, should also be determined.

It is biologically plausible that a combined ALB+PZQ schedule may improve clearance of viable cysts and thus provide better cysticidal efficacy as a consequence of a higher concentration of both active metabolites. 

Given the relative scarcity of clinical trials, more comparative interventional studies,especially randomized controlled trials in long-term clinical evolution, are required in order to clarify the controversy over the validity of parasitic therapy in patients with NCC.

Given the risks and fallibility of anti cysticercal therapy, only the implementation of control measures will be able to eliminate this serious public health problem.

## PREVENTIVE MEASURES

By the first part of 20th Century,*T. solium*infections had been almost eliminated in Europe. This process took place over several decades and required many changes in economic, educational and sanitary standards, and improvement in the effectiveness of medical and veterinary services, especially meat inspection. These are not likely to be duplicated soon in many parts of the developing world. Therefore, the realistic aim of control is to reduce the incidence and prevalence of*T. solium*infections in humans and pigs to the level that human NCC does not constitute a major public health and economic problem in a given endemic area^77^.

The control and elimination of *T. solium*/cysticercosis is hindered by many factors, including the lack of reliable epidemiological data on infection. No national surveillance or control program is currently in place, except in China, despite the endemicity of *T. solium* and epilepsy in low-and-middle-income countries^1^. 

New cases can be prevented with health and educational community interventions^78^ ^-^ ^80^ and a One Health approach involving^1^:

- vaccination and anthelmintic treatment of pigs to prevent infection with *T. solium* cysticerci;
- improved pig management practices to prevent exposure of pigs to human feces;
- improved sanitation to prevent contact between pigs and humans and *T. solium* eggs in human feces and in the environment;
- meat inspection and sufficient cooking of pork to reduce the risk of humans becoming infected;
- treatment of human taeniasis; and
- health education to promote hand hygiene, food safety, sanitation and pig management^1^.

Considering the differences in cultures, levels of education, socio-economic levels, and sanitary conditions, etc. these control measures should be adapted to the local epidemiological situation. The prevention strategies must rely on multiple approaches, tailoring each to the specific features of the particular endemic area^77^.

In 1992, a pilot project was launched in Ribeirão Preto, São Paulo, Brazil. The project included a number of environmental sanitation measures, compulsory notification, meat inspection, monitoring of vegetable crops and commercial concerns, and active surveillance of taeniasis among food handlers^78^ ^-^ ^80^.

In conclusion, NCC is the most common cause of acquired epilepsy worldwide, especially in endemic areas. 

Clinical manifestations of NCC are pleomorphic depending upon the number, type, size, location and stage of cysticerci development, as well as on the host immune response, and there is not a pathognomonic manifestation.

The diagnosis of NCC is based on neuroimaging studies (CT and MRI), immunodiagnostic tests in serum (EITB) and CSF (antibodies and/or antigens) that are not readily available in many settings where NCC is prevalent due to resource constraints.

Therapy for NCC has advanced with the advent of two drugs that are considered to be effective: PZQ and ALB. Most comparative studies have shown that ALB is more effective than PZQ in inducing overall clinical improvement. Nowadays, ALB is the medication of choice for the treatment of symptomatic patients presenting multiple viable cysts in the brain parenchyma on neuroimaging studies. However, anti cysticercal therapy has been marked by an intense controversy and the benefit of this treatment has been questioned. Randomized controlled trials evaluating the clinical benefit have yielded conflicting results with some studies indicating a benefit and others failing to show a difference. The discrepant data over the efficacy of anti cysticercal therapy may be explained by high inter-individual variability in plasma concentration of ALB sulfoxide (ASOX), the active metabolite of ALB, and the complex interactions of several drugs with ASOX. 

There are several interventions that can be implemented for the control of *T. solium*, and a One-Health approach is the most effective, efficient, and sustainable control. However, implementation of control measures has been limited due to a variety of reasons, including the lack of appropriate tools. Over the last few years, a new set of tools has been developed to assist health care providers in appropriate evidence-based management of NCC, and so assist public health stakeholders in implementing control measures for *T. solium*.
